# Leukocyte Telomere Length in Relation to 17 Biomarkers of Cardiovascular Disease Risk: A Cross-Sectional Study of US Adults

**DOI:** 10.1371/journal.pmed.1002188

**Published:** 2016-11-29

**Authors:** David H. Rehkopf, Belinda L. Needham, Jue Lin, Elizabeth H. Blackburn, Ami R. Zota, Janet M. Wojcicki, Elissa S. Epel

**Affiliations:** 1 Department of Medicine, Stanford University, Stanford, California, United States of America; 2 Department of Epidemiology, University of Michigan, Ann Arbor, Michigan, United States of America; 3 Department of Biochemistry and Biophysics, University of California San Francisco, San Francisco, California, United States of America; 4 Department of Environmental and Occupational Health, George Washington University, Washington, DC, United States of America; 5 Department of Pediatrics, University of California San Francisco, San Francisco, California, United States of America; 6 Department of Psychiatry, University of California San Francisco, San Francisco, California, United States of America; University of Oxford, UNITED KINGDOM

## Abstract

**Background:**

Leukocyte telomere length (LTL) is a putative biological marker of immune system age, and there are demonstrated associations between LTL and cardiovascular disease. This may be due in part to the relationship of LTL with other biomarkers associated with cardiovascular disease risk. However, the strength of associations between LTL and adiposity, metabolic, proinflammatory, and cardiovascular biomarkers has not been systematically evaluated in a United States nationally representative population.

**Methods and Findings:**

We examined associations between LTL and 17 cardiovascular biomarkers, including lipoproteins, blood sugar, circulatory pressure, proinflammatory markers, kidney function, and adiposity measures, in adults ages 20 to 84 from the cross-sectional US nationally representative 1999–2002 National Health and Nutrition Examination Survey (NHANES) (*n* = 7,252), statistically adjusting for immune cell type distributions. We also examine whether these associations differed systematically by age, race/ethnicity, gender, education, and income. We found that a one unit difference in the following biomarkers were associated with kilobase pair differences in LTL: BMI -0.00478 (95% CI -0.00749–-0.00206), waist circumference -0.00211 (95% CI -0.00325–-0.000969), percentage of body fat -0.00516 (95% CI -0.00761–-0.0027), high density lipoprotein (HDL) cholesterol 0.00179 (95% CI 0.000571–0.00301), triglycerides -0.000285 (95% CI -0.000555–-0.0000158), pulse rate -0.00194 (95% CI -0.00317–-0.000705), C-reactive protein -0.0363 (95% CI 0.0601–-0.0124), cystatin C -0.0391 (95% CI -0.0772–-0.00107). When using clinical cut-points we additionally found associations between LTL and insulin resistance -0.0412 (95% CI -0.0685–-0.0139), systolic blood pressure 0.0455 (95% CI 0.00137–0.0897), and diastolic blood pressure -0.0674 (95% CI -0.126–-0.00889). These associations were 10%–15% greater without controlling for leukocyte cell types. There were very few differences in the associations by age, race/ethnicity, gender, education, or income. Our findings are relevant to the relationships between these cardiovascular biomarkers in the general population but not to cardiovascular disease as a clinical outcome.

**Conclusions:**

LTL is most strongly associated with adiposity, but is also associated with biomarkers across several physiological systems. LTL may thus be a predictor of cardiovascular disease through its association with multiple risk factors that are physiologically correlated with risk for development of cardiovascular disease. Our results are consistent with LTL being a biomarker of cardiovascular aging through established physiological mechanisms.

## Abstract 2

David Rehkopf and colleagues analyze the association of leukocyte telomere length with cardiovascular disease risk biomarkers.

## Author Summary

**Why Was This Study Done?:**

- Leukocyte telomere length (LTL) is a biomarker of white blood cell division and is strongly associated with age.
- There are still mixed findings on how LTL is related to different types of mortality.
- Observational evidence and quasi-experimental studies suggest that there is an association between shorter LTL and cardiovascular disease mortality.
- It is unclear, however, whether or not LTL is completely independent of already known biological pathways of cardiovascular disease.

**What Did the Researchers Do and Find?:**

- We examined US nationally representative data on participants ages 25 to 84 that had LTL measured along with 17 other cardiovascular risk biomarkers.
- We found associations between LTL and measures of adiposity (BMI, waist circumference, percentage of body fat), along with C-reactive protein, cystatin C, high-density lipoprotein (HDL) cholesterol, triglycerides, insulin resistance, systolic blood pressure, diastolic blood pressure, and pulse rate.
- We did not find that there were a meaningful number of differences in these relationships by age, race/ethnicity, or socioeconomic position, suggesting that the associations we find are generally consistent across different population groups.

**What Do These Findings Mean?:**

- Our findings suggest that LTL is associated with cardiovascular risk factors across different physiological systems.
- The study findings do not have any implications for whether LTL is a cause of cardiovascular disease.

## Introduction

Shorter leukocyte telomere length (LTL) is significantly associated with increased risk of cardiovascular disease, irrespective of adjustment for conventional risk factors [1]. A meta-analysis of 24 studies with 43,725 participants and 8,400 patients with cardiovascular disease estimated a relative risk of 1.54 (95% CI 1.30–1.83), comparing those with the longest to shortest third of LTL [1]. Although these finding are consistent with a role for LTL in cardiovascular disease etiology, the critical details of how LTL is related to known metabolic pro-inflammatory markers and cardiovascular risk factors in the pathway to cardiovascular disease are unclear [2]. It is thought that telomere length shortens in relationship to multiple types of biochemical stressors and thus may represent a cumulative index of known risk factors. Our study will contribute to answering this question using a large US nationally representative sample with a comprehensive number of biomarkers measured along with LTL. Examining the relationship of telomere length with multiple biomarkers concurrently will inform us of whether LTL might be a marker for one primary risk factor, such as inflammation, or of multiple risk factors. This analysis will thus help to better understand whether LTL represents a unique biomarker that is independent from other known risk factors for cardiovascular disease.

In previous research, LTL was associated with myocardial infarction and stroke among younger (≤73 years) individuals [3]; rate of LTL shortening was associated with cardiovascular disease among elderly men [4]; LTL was associated with congestive heart failure [5]; and LTL was associated with prospective cardiovascular disease events [6]. Single nucleotide polymorphisms (SNPs) that are associated with telomere length are also associated with cardiovascular disease, offering a complementary source of evidence consistent with the observational data [7]. There are several prior studies that have each examined cardiovascular risk biomarkers and their association with LTL. The most comprehensive analysis to date examined approximately 2,500 community-dwelling white individuals from Belgium aged 35–55 who did not have diagnosed cardiovascular disease [8]. They found very little correlation with traditional lipid biomarkers but did find associations with markers of inflammation, including C-reactive protein. Other studies have found mixed associations between diastolic blood pressure and LTL, with one study finding an inverse association [3] and another no association [4]. A prior study of a nationally representative sample of 1,000 individuals in Costa Rica found a statistically significant positive association with systolic blood pressure [9].

To our knowledge, this is the first study to comprehensively examine the relationships between cardiovascular risk factor biomarkers and LTL in a large, US nationally representative sample, allowing us to either confirm or refute the results of recent studies on telomere length in relation to specific metabolic, immune function, and cardiovascular biomarkers from smaller, isolated populations and to perform analyses controlling for multiple measures of socioeconomic position, clinical variables, and chronic disease as potentially confounding variables. In addition, because we have a nationally representative study that oversampled racial/ethnic minority populations, we can also appropriately test for effect measure modification by age, race/ethnicity, gender, education, and income. This is critical, as a prior meta-analysis suggested that there may be substantial heterogeneity across demographic groups in the relationship between LTL and myocardial infarction but this study did not have enough data to robustly test this hypothesis [10]. A further contribution of our work is examining these associations among individuals of all ages, because an important potential contribution of LTL is using it as a biomarker of aging across the life course. Prior studies have focused primarily on relationships with cardiovascular biomarkers in the population aged 65 and older [11]. In addition, the use of a wide range of ages is important because it allows for analyses conditional on current chronic disease, which is much more common among older individuals.

Because LTL comes from a mix of blood types that have systematically different extents of differentiation depending on function and thus have different mean telomere lengths, previously demonstrated associations of LTL and health outcomes may be confounded by cell type composition [12]. We thus also examine associations statistically controlling for cell type composition. Finally, given the possibility that a linear model may not be the appropriate functional form for examining the association between LTL and continuous cardiovascular biomarkers, we present sensitivity analyses fit with penalized spline models and also examine associations with clinical cut-points of biomarkers.

## Materials and Methods

### Study Population

We pooled data from the 1999–2000 and 2001–2002 cycles of National Health and Nutrition Examination Survey (NHANES), a nationally representative survey and physical examination of the civilian, noninstitutionalized US population conducted by the US Centers for Disease Control and Prevention (CDC). All data that we used for the analyses presented here are available for public download (http://www.cdc.gov/nchs/nhanes/nhanes_questionnaires.htm). Between 1999 and 2002, DNA samples were stored from NHANES participants aged 20 and older to establish a national probability sample of genetic material for future research. Our analysis is limited to these years of data because of the availability of DNA for LTL assay during only these years. We excluded individuals aged 85 and older from our analysis because of mortality selection over this age and because most traditional risk factors for disease are not predictive of morbidity and mortality in this population. Of the 9,191 participants aged 20–84 in the sample who were eligible to provide DNA, 7,525 (82%) provided DNA, consented to its use in future research, and had a sufficient quality and quantity of DNA to estimate telomere length. Non-Hispanic blacks, women, and subjects older than 60 years of age were less likely to give consent for future genetic research [13]. Participants provided written consent for participation in this study, and all study protocols were approved by the Institutional Review Board at the CDC.

### LTL Measurements

Aliquots of purified DNA were provided by the National Center for Health Statistics. DNA was isolated from whole blood using the Puregene (D-50K) kit protocol (Gentra Systems, Inc., Minneapolis, Minnesota) and stored at -80°C. To measure mean LTL, quantitative PCR assay was used to determine the relative ratio of telomere repeat copy number to single-copy gene copy number (*T/S* ratio), with the full details of this standard protocol described elsewhere [14,15]. Each sample was assayed three times on three different days. The samples were assayed on duplicate wells, resulting in six measurements per sample. Sample plates were assayed in groups of three plates, and no two plates were grouped together more than once. Each assay plate contained 96 control wells. Any assay runs with eight or more invalid control wells were considered a failed run and were excluded from further analysis (<1% of runs). The mean of the *T/S* ratio values was calculated, and the largest and the smallest *T/S* ratio values in the set were marked as potential outliers. Then the mean of the *T/S* ratio value was calculated without the potential outliers. If the absolute value of the log of the ratio between the recalculated mean (excluding the potential outliers) to the value of the potential outlier was greater than 0.4, then the value was marked as an outlier (98.7% of all samples contained no outliers). The LTL assay lab was blind to the sample characteristics. The inter-assay coefficient of variability for LTL was 4%. The equation for conversion from T/S ratio to kb pairs used was 3,274 + 2,413 * (T/S) / 1,000 [16]. This conversion ratio is likely to differ between laboratories and even between assays within the same lab; thus, exact kb pair values we report should be used as an approximation of actual telomere length. Although this does not impact the internal validity of the analyses presented here, the absolute level of base pair length should not be used in direct comparison to other studies. The assays were conducted in the Blackburn Laboratory at the University of California, San Francisco. Due to the extreme right skew (high values) of a small number of observations that are likely to be assay errors and not biologically plausible, we excluded the top 1% of measures (*n* = 76) from all of our analyses.

### Blood Sample Characteristics

**Fig 1:**
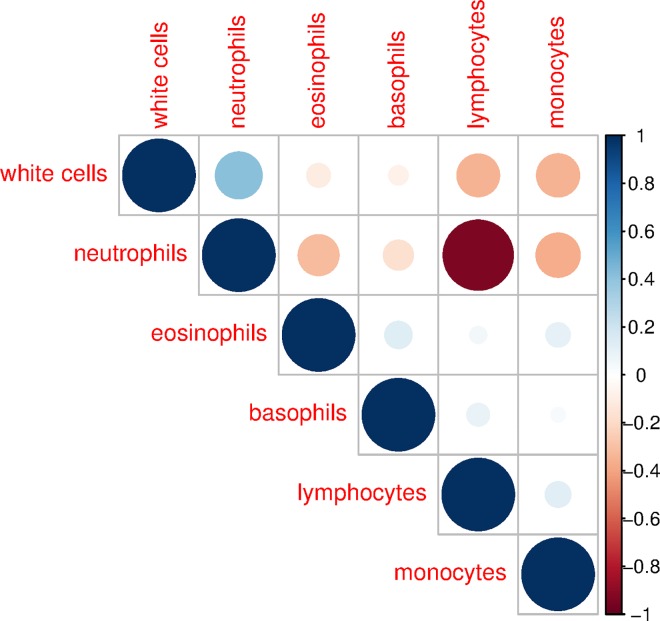
Correlation structure of blood constituent cell types, ages 20–84, NHANES 1999–2002. The scale to the right of the figure indicates the strength of correlation, with darker and larger dots indicating stronger correlation. The six constituent blood components are ordered by hierarchical clustering.

NHANES 1999–2002 measured a number of characteristics of the blood samples from which DNA was extracted. These characteristics included the following: white blood cells (SI), lymphocytes (%), monocytes (%), neutrophils (%), eosinophils (%), and basophils (%). Overall white blood cell count may act as a confounder because it can capture whether infection is occurring, which may have resulted in proliferation of additional new cells within each cell type, which may have different telomere lengths, and which may not be captured by the five cell type composition measures. Fig 1 shows the correlation structure between these six factors. The only strong correlation is between neutrophils and lymphocytes. This suggests that most of these measures are capturing unique information about the blood component cell type composition.

### Biomarker Outcomes Examined

**Fig 2:**
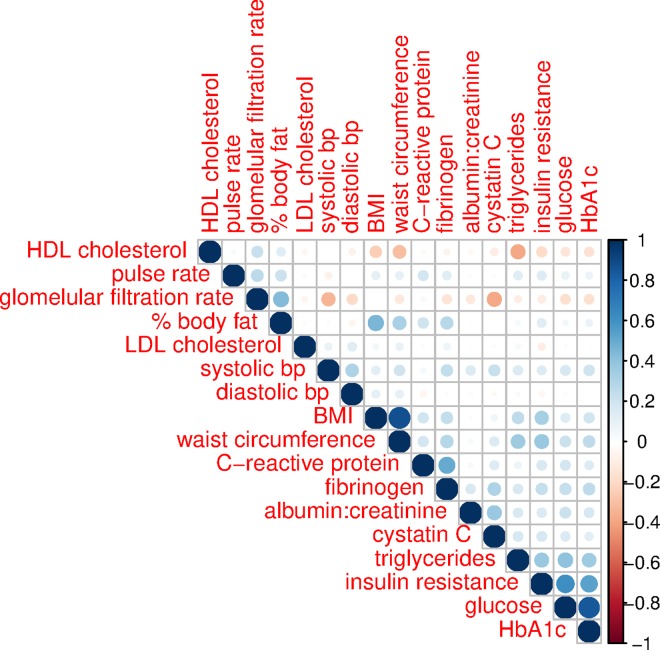
Correlation structure of biomarkers, ages 20–84, NHANES 1999–2002. The scale to the right of the figure indicates the strength of correlation, with darker and larger dots indicating stronger correlation. The 17 biomarker components are ordered by hierarchical clustering.

NHANES 1999–2002 allows for an examination of 17 factors within six categories of biomarkers that are risk factors for cardiovascular disease: lipoproteins (high-density lipoprotein [HDL] cholesterol, low density lipoprotein [LDL] cholesterol, triglycerides), blood sugar (glucose, hemoglobin A1c, insulin resistance), circulatory pressure (systolic blood pressure, diastolic blood pressure, pulse rate), proinflammatory (C-reactive protein, fibrinogen), kidney function (cystatin C, glomerular filtration rate, urine albumin to creatinine ratio [ACR]), and adiposity measures (body mass index [BMI], waist circumference, estimated percentage of body fat). The details of the measurement of these factors have been described in the NHANES documentation [17]. We used values of serum creatinine to estimate the glomerular filtration rate using the reexpressed four-variable MDRD Study equation (glomerular filtration rate = 175 × standardized (S_cr_)^−1.154^ × (age)^−0.203^) [18] without equivalization for race/ethnicity and gender (we statistically controlled for race/ethnicity and gender in our analyses). The glomerular filtration rate is presented as mL/min/1.73 m^2^. Insulin resistance was measured by HOMA-IR, which was calculated as the product of fasting glucose (mmol/L) and fasting insulin (μU/ML) divided by 22.5. Urine albumin creatinine ratio was calculated as urine albumin divided by urinary creatinine and reported as mg/g. Unless otherwise indicated, biomarkers are reported in SI units. Fig 2 shows the correlation structure of these 17 measures, ordered by a hierarchical clustering algorithm. Although we see an expected level of correlation between measures of adiposity, including BMI and waist circumference as well as hemoglobin A1c and glucose, most other measures are not substantially correlated (>0.6). This suggests that it is appropriate to examine this wide range of biomarkers that capture generally unique aspects of physiology within several different systems. Because the health risks of levels of each of these biomarkers may in some cases be nonlinear, we also examined each as a clinical threshold for a high-risk level of the biomarker. We used the following levels as indicating an increased level of clinical risk based on ATP III guidelines and other relevant literature: HDL cholesterol (<40 mg/dl) [19], LDL cholesterol (≥160 mg/dl) [19], triglycerides (≥200 mg/dl) [19], glucose (>100 mg/dl) [20], hemoglobin A1c (>5.4%) [21], HOMA-IR (≥2.5) [22], systolic blood pressure (≥140 mm Hg) [23], diastolic blood pressure (≥90 mm Hg) [23], pulse rate (≥83 beats per minute) [24,25], C-reactive protein (>3.0 mg/L) [26], fibrinogen (>4.0 g/mL) [27], cystatin C (>1.29 mg/L) [28], estimated glomerular filtration rate (<60 mL/min/1.73 m^2^) [29], urine ACR (>30 mg/g) [30], BMI (≥30 kg/m^2^) [31], waist circumference (>102 cm for men, >88 cm for women) [31], and estimated percentage of body fat (>25% for men, >30% for women) [32]. We also examine a summary measure of cardiometabolic risk—the metabolic syndrome, which is a sum of whether a participant is above the clinical cut-point for waist circumference, triglycerides, HDL cholesterol, systolic blood pressure, and glucose. Individuals with a score of 3 or more are considered to have the metabolic syndrome [33].

### Demographic Variables

All demographic variables that we use in our analysis are derived from respondent self-report, including race/ethnicity (white, black, Mexican American, and other), marital status (married or living with partner versus not), place of birth (outside of the US or within the US), level of educational attainment (less than high school, high school, more than high school), and occupation (white collar and professional; white collar, semi-routine; blue collar, high skill; blue collar, semi-routine; not working). The occupation measure was based on the self-reported type of work that was done the longest, using a previously described classification [34]. Our measure of income was the NHANES calculated poverty–income ratio (PIR), or the ratio of household income to poverty threshold adjusted for family size and inflation and used as a continuous measure.

### Health and Health Behaviors

All health and health behavior measures were derived from self-reported data. Respondents were asked separate questions on whether they had “ever been told by a doctor or other health professional that” they had high blood pressure or hypertension, had diabetes, or had congestive heart failure, coronary heart disease, angina, angina pectoris, a heart attack, or a stroke. Participants were also asked if they had ever been told of having “weak or failing kidneys? Do not include kidney stones, bladder infections, or incontinence.” Participants were asked whether they had smoked at least 100 cigarettes in their entire life and whether they currently smoke cigarettes, which we label as ever smoked and current smoker. Participants were also asked whether they had participated in “moderate activities that cause only light sweating or a slight to moderate increase in breathing or heart rate. Some examples are brisk walking, bicycling for pleasure, golf and dancing” over the past 30 days, as well as whether they had participated in “vigorous activities for at least ten minutes that cause heavy sweating or large increases in breathing or heart rate? Some examples are running, lap swimming, aerobics classes, or fast bicycling” at least once over the last 30 days.

### Missing Data

Of the 7,525 individuals in the analytic sample, there were the following numbers of individuals missing demographic, health behavior, and chronic disease variables: education (1), income (660), occupation (3,072), marital status (358), current smoker (14), vigorous physical activity (6), hypertension (75), and diabetes (3). We also imputed missing data for cardiovascular biomarkers that were missing the following number of observations: HDL cholesterol (6), LDL cholesterol (4,217), triglycerides (4,084), glucose (4,086), insulin (3,831), hemoglobin A1c (5), systolic blood pressure (241), diastolic blood pressure (241), pulse pressure (206), C-reactive protein (1), fibrinogen (2,783), cystatin C (3,711), glomerular filtration rate (4,086), urine albumin to creatinine ratio (100), BMI (197), waist circumference (224), and percentage of body fat (4,672). Note that samples were intentionally smaller for LDL cholesterol, triglycerides, glucose, and insulin because we examine these only in the subsample that fasted overnight, and special survey sample weights for this subsample are used. The estimated body fat from bioimpedance spectroscopy has a larger number of missing observations because this was only performed on participants up to the age of 49 years old. Cystatin C was measured only on participants aged 60 and above and on a 25% random sample of participants aged 12–59 years old. Fibrinogen was assayed only on individuals aged 40 and older. In order to avoid the bias caused by complete case analysis, we imputed missing variables for the demographic control variables and cardiovascular disease biomarkers using a nonparametric missing value imputation with Random Forest, implemented with the R package missForest [35] fit with 100 trees. This approach has several advantages for our particular study as compared to multiple imputation methods for missing data, including that it can be used with specialized survey functions and nonlinear models. This method of imputing missing data has been shown to compare favorably, with the missForest algorithm having the smallest prediction difference between the imputed and non-missing data sets [36]. The large number of missing observations for occupation mean that there will be some residual confounding to measurement error in the imputed data for occupation, but this approach is preferable to the larger degree of uncontrolled confounding that would exist if excluding occupation from the models [37]. Note that based on prior empirical work in the literature we did not impute missing values of our dependent variable, LTL, as this generally results in either no improvement in model accuracy or more biased results [38,39].

### Statistical Analysis

Analyses were conducted in R using the survey package [40], with models fit using the svyglm() function that uses inverse probability weighting and design-based standard errors All analyses accounted for the clustered sampling design and the examination survey weights as required for appropriate analysis of this data [41]. For variables that were only measured for individuals who participated in an overnight fast (LDL cholesterol, triglycerides, glucose, insulin), fasting examination survey weights were used.

We used multivariable regression models to assess the relationship between LTL and each biomarker. Consistent with most prior literature on biomarkers and telomere length, we examined telomere length as a linear variable.

For all analyses, we present a model 1 that shows the coefficient from a simple linear regression model for the cell sample characteristic or cardiovascular biomarker in relation to the dependent variable of LTL. In model 2, we fit a multiple linear regression model that included additional variables that were demonstrated by Needham et al. (2013) to be predictors of LTL in this study population: race/ethnicity (indicators for white, Mexican American, black, and other), education (indicators for less than high school and high school diploma, with some college or more as the omitted comparison category), income, occupation (white collar and professional; white collar, semi-routine; blue collar, high skill; blue collar, semi-routine; not working), married, foreign born, age (as continuous linear), and age-squared. The use of continuous linear age along with an age-squared term is important for analyses of LTL given the strength with which it is associated with age and the potential for nonlinearity in this association [42]. Model 3 additionally includes two measures of smoking and two measures of physical activity, as these may act as confounding factors of the relationship between LTL and cardiovascular biomarkers. We fit this as a separate model because we want to assess whether the associations observed between biomarkers and LTL are attenuated due to health-related behaviors that are associated with LTL. Model 4 additionally includes the six available cell type composition measures. We fit each of the above models both with continuous levels of the biomarkers and with the two category clinical risk threshold measures. We also performed sensitivity analysis, restricting the population to individuals that did not have chronic disease risk, in order to test whether our results for the general population also apply to a health population. In particular, these robustness checks of results allow us to address the possible limitation that the presence of chronic disease may cause associations between LTL and cardiovascular disease risk biomarkers.

We a priori specified that we would test for effect measure modification between the continuous measures of the biomarkers and LTL by gender, age (20–44 and 65–84), income (PIR), education (less than high school), and race/ethnicity (black and Mexican American) and report the magnitude of differences observed and the 95% CIs when interaction terms did not include the null of no association.

Finally, given that there are unclear priors on the best functional form to model the relationship between each of these biomarkers and LTL, we conducted sensitivity analysis using a penalized spline model that allows there to be a nonlinear relationship between dependent and independent variables. These models were fit using a generalized additive mixed model that fits a number of splines across the data then uses a penalized fitting algorithm to fit a number of slopes that best fit the data. We specified a maximum of four degrees of freedom for the spline basis, with the best fitting model fit by generalized cross validation [43].

## Results

### Sample Characteristics

**Fig 3:**
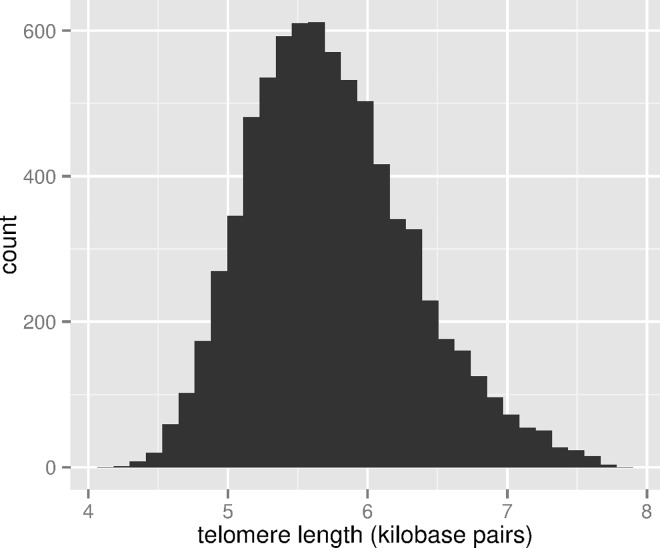
Distribution of LTL (kb pairs) in the sample, ages 20–84, NHANES 1999–2002.

Table 1 shows the weighted distribution of population demographic characteristics for the full NHANES 1999–2002 population aged 20–84, the population with measured telomere length, as well as the characteristics for the population with measured telomere length for which missing values were imputed. For the full NHANES population, the distribution of these factors reflects their distribution in the US noninstitutionalized population consistent with the sampling design of the NHANES study. The population with measured telomere length differs primarily because of who consented to the future use of their DNA. Although the populations are very similar, there are about 1% fewer black participants in the sample with telomere length, as well as fewer foreign-born individuals. The distributions by education, income, and occupation are similar. The sample distributions for the final imputed sample only differ notably for occupational classification due to the large number of missing responses for this question. Fig 3 shows the distribution of LTL.

**Table 1:** Sample characteristics, ages 20–84, NHANES 1999–2002.

	Full NHANES (*n* = 9,191)	With Telomere Length (*n* = 7,252)	Final Imputed (*n* = 7,252)
	Proportion/mean	Proportion/mean	Proportion/mean
Age (mean)	45.5	45.7	45.7
Female	0.52	0.511	0.511
**Race/ethnicity**			
Black	0.108	0.093	0.093
White	0.707	0.727	0.727
Mexican American	0.071	0.071	0.071
Other race/ethnicity	0.113	0.109	0.109
**Education**			
Less than high school	0.216	0.212	0.212
High school	0.256	0.26	0.26
Some college	0.526	0.527	0.527
**Poverty: Income ratio (mean)**	2.98	3.02	3.00
**Occupation**			
White collar—high	0.241	0.247	0.412
Blue Collar—high	0.112	0.115	0.115
White collar—low	0.223	0.222	0.175
Blue collar—low	0.392	0.384	0.263
No work	0.033	0.032	0.029
**Foreign born**	0.154	0.147	0.148
**Married**	0.664	0.660	0.664
**Smoked (ever)**	0.497	0.503	0.503
**Smoker (current)**	0.247	0.248	0.248
**Physical activity (moderate)**	0.472	0.484	0.484
**Physical activity (vigorous)**	0.351	0.358	0.358
**Kidney disease**	0.021	0.021	0.021
**Hypertension**	0.254	0.255	0.255
**Diabetes**	0.066	0.066	0.066
**Cardiovascular disease**	0.08	0.08	0.08

### LTL and Constituent Cell Types

Table 2 shows coefficients of association between cell types comprising the blood sample from which DNA was extracted and the LTL assayed from the leukocytes. The model-based coefficients in the table can be interpreted as the difference in 1 kb pair of LTL associated with a one unit change in the cell type. For example, the coefficient of 0.0272 for Basophils in model 1 means that a difference in 1 kb of telomere length is associated with 2.72% difference in Basophils. Model 1 presents the unadjusted correlation coefficients. Model 2 includes social and demographic factors. Model 3 additionally controls health behaviors. In the unadjusted model 1, monocytes and eosinophils were negatively associated with LTL, and lymphocytes were positively associated, with 95% CIs that did not include 0. After controlling for demographic factors, the concentration of white blood cells is significantly associated with LTL. The results from model 3 are similar, for which a higher level of white blood cells is associated with lower LTL. We also examined this model in a restricted population of individuals aged 25 to 84 who did not have kidney disease, hypertension, diabetes, or cardiovascular disease, and associations were either similarly close to 0 or the magnitude of the association did not differ by more than 10% (S1 Table).

**Table 2:** Association of LTL with concentration of white blood cells and constituent cell types, ages 20 to 84, NHANES 1999–2002.

	Model 1—unadjusted			Model 2—demographic adjusted			Model 3—demographic + health-related behaviors		
	coef	95% CI		coef	95% CI		coef	95% CI	
White blood cells (SI)	-0.00984	-0.0219,	0.00224	-0.0161**	-0.0264,	-0.00576	-0.0152*	-0.0253,	-0.0051
Lymphocytes (%)	0.00288*	0.0000759,	0.00568	0.0000529	-0.00229,	0.0024	-0.000106	-0.00241,	0.0022
Monocytes (%)	-0.0126*	-0.0217,	-0.00358	-0.00136	-0.00985,	0.00713	-0.00197	-0.0105,	0.0066
Neutrophils (%)	-0.00109	-0.00355,	0.00136	0.000169	-0.0019,	0.00224	0.000354	-0.00168,	0.00239
Eosinophils (%)	-0.00989**	-0.016,	-0.00376	-0.00433	-0.0103,	0.00167	-0.00468	-0.0107,	0.0013
Basophils (%)	0.0272	-0.0264,	0.0807	0.0519	-0.000992,	0.105	0.0493	-0.00445,	0.103

Sample size for all analyses is *n* = 7,252. The dependent variable in all models is LTL (in kbp). Each line shows coefficients and 95% CIs from separate statistical models. Model 1 does not adjust for any covariables. Model 2 adjusts for the following covariables: race/ethnicity (white, Mexican American, black, and other), gender, foreign birthplace, education (less than high school, high school diploma, more than high school), class of work (white collar high, blue collar high, white collar low, blue collar low, no work), income, marital status (married or living with partner), age (as continuous), and age-squared. Model 3 additionally controls for ever smoked, current smoker, moderate physical activity, and vigorous physical activity.

*p < 0.05

**p < 0.01

We also examined interactions with gender, race/ethnicity (black and Mexican American), education (less than high school diploma), income (continuous), and age (25–44 and 65–84)—a total of 42 interactions (S2 Table). We find a more negative association for monocytes for black participants (interaction coefficient = -0.0202; 95% CI -0.0336–-0.00684), a more positive association of eosinophils with higher income (interaction coefficient = 0.00469; 95% CI 0.00149–0.00789), and both lower lymphocytes (interaction coefficient = -0.00348; 95% CI -0.00657–-0.000391) and higher neutrophils (interaction coefficient = 0.00290; 95% CI 0.000418–0.00539) among participants age 65 and older. We thus found four interactions for which the null of 0 was not included in the confidence interval when 2–3 would be expected by chance alone.

### LTL and Biomarkers

**Fig 4:**
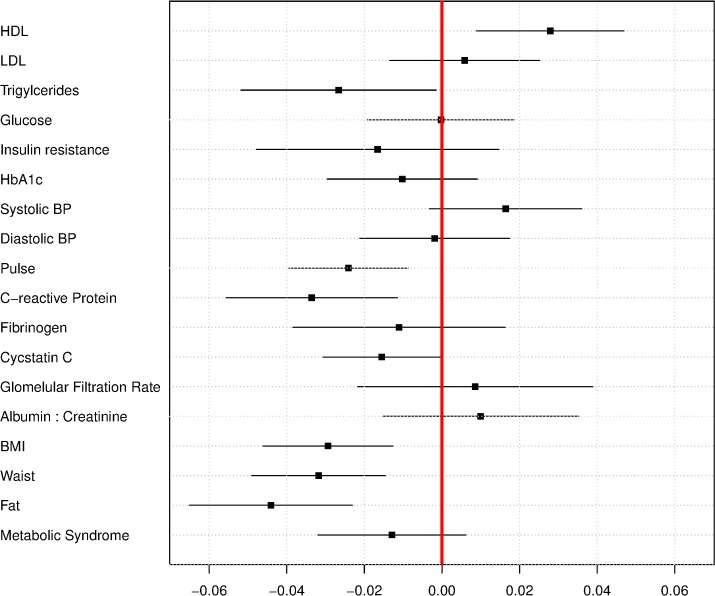
Regression coefficients for relationship between biomarkers and LTL, in standard deviations of biomarker per kb of LTL, ages 20–84, NHANES 1999–2002.

Table 3 presents coefficients of the association between biomarkers and LTL in the categories of lipoproteins, blood sugar, circulatory pressure, proinflammatory, kidney function, and adiposity measures. There were associations with LTL for 13 of the 17 biomarkers examined in the unadjusted models (model 1) for which the null association of 0 was not within the estimated 95% CIs, with eight of these associations remaining after controlling for demographic factors, health behaviors, and cell type (model 4). These factors were HDL cholesterol, triglycerides, pulse rate, C-reactive protein, cystatin C, BMI, waist circumference, and percentage of body fat. There was no association with the summary measure of metabolic syndrome after controlling for demographic characteristics, nor when additionally controlling for health-related behaviors. There was generally between a 10%–15% reduction in the magnitude of the coefficient after controlling for cell type (comparing models 2 and 3 to model 4), consistent with the fact that there may be some confounding of the associations we observed in models 2 and 3 by cell type. Fig 4 shows a dot plot of coefficients and 95% CIs comparing the magnitudes of association with LTL across each of the 17 biomarkers and the summary measure of the metabolic syndrome. The coefficients are from the same model as model 4, but the biomarkers are standardized (by dividing by their standard deviation), so the magnitude of the associations with LTL can be directly compared to each other. The scale of interpretation is how much of a standard deviation of the biomarker is associated with a 1 kb pair difference in LTL. The strongest negative correlations were for percentage of body fat, BMI, C-reactive protein, and waist circumference, followed by pulse. The strongest positive correlation was for HDL, which was of a similar magnitude of association as pulse rate. Note that HDL is the only biomarker we examined where higher levels are associated with lower cardiovascular disease risk [44].

**Table 3:** Regression of LTL on continuous biomarkers, ages 20 to 84, NHANES 1999–2002.

	Model 1–unadjusted			Model 2–demographic adjusted			Model 3–demographic + health-related behaviors			Model 4–demographic + health-related behaviors + cell type adjusted		
	coef	95% CI		coef	95% CI		coef	95% CI		coef	95% CI	
**Lipoproteins**												
HDL cholesterol	0.000941	-0.000445,	0.00233	0.00212**	0.000906,	0.00334	0.00206**	0.000839,	0.00328	0.00179*	0.000571,	0.00301
LDL cholesterol	-0.000963*	-0.00186,	-0.0000644	0.000271	-0.000491,	0.00103	0.0000858	-0.000601,	0.000773	0.000238	-0.000547,	0.00102
Triglycerides	-0.000946***	-0.00135,	-0.000546	-0.00035*	-0.000642,	-0.0000577	-0.000192	-0.000421,	0.0000383	-0.000285*	-0.000555,	-0.0000158
**Blood sugar**												
Glucose	-0.00281***	-0.00374,	-0.00187	-0.0000894	-0.000721,	0.000542	-0.000266	-0.000835,	0.000304	-0.00000737	-0.000604,	0.000589
Insulin resistance	-0.0135**	-0.0229,	-0.00421	-0.00483	-0.0117,	0.00203	-0.00309	-0.0063,	0.000114	-0.00324	-0.00934,	0.00286
HbA1c	-0.0765***	-0.0951,	-0.0578	-0.014	-0.0312,	0.00326	-0.0138	-0.0312,	0.00365	-0.00951	-0.0274,	0.00843
**Circulatory pressure**												
Systolic blood pressure	-0.00553***	-0.00659,	-0.00448	0.000703	-0.000243,	0.00165	0.000732	-0.000219,	0.00168	0.000815	-0.000158,	0.00179
Diastolic blood pressure	0.0000221	-0.00137,	0.00142	-0.0000155	-0.00139,	0.00136	-0.0000635	-0.00143,	0.0013	-0.000138	-0.00154,	0.00126
Pulse rate	-0.000143	-0.00151,	0.00123	-0.00248**	-0.00375,	-0.00121	-0.00226**	-0.00352,	-0.00101	-0.00194*	-0.00317,	-0.000705
**Pro-inflammatory**												
C-reactive protein	-0.0699***	-0.101,	-0.039	-0.0453**	-0.0693,	-0.0213	-0.043**	-0.0671,	-0.0188	-0.0363*	-0.0601,	-0.0124
Fibrinogin	-0.117***	-0.154,	-0.081	-0.0297	-0.0718,	0.0124	-0.0263	-0.0671,	0.0145	-0.0154	-0.0535,	0.0227
**Kidney function**												
Cystatin C	-0.273***	-0.368,	-0.178	-0.051*	-0.0942,	-0.00771	-0.0474*	-0.0878,	-0.00695	-0.0391*	-0.0772,	-0.00107
Glomerular filtration rate	0.00227***	0.0019,	0.00264	0.00000239	-0.000554,	0.000559	0.0000848	-0.000464,	0.000633	0.000158	-0.000402,	0.000719
Albumin: Creatinine	-0.0000444	-0.000107,	0.0000177	0.0000192	-0.0000392,	0.0000776	0.0000203	-0.0000375,	0.000078	0.0000221	-0.0000341,	0.0000782
**Adiposity**												
BMI	-0.00752***	-0.0107,	-0.00432	-0.00545**	-0.00808,	-0.00282	-0.00552**	-0.0083,	-0.00275	-0.00478*	-0.00749,	-0.00206
waist circumference	-0.00538***	-0.00676,	-0.00399	-0.00247**	-0.00362,	-0.00132	-0.00242**	-0.0036,	-0.00124	-0.00211*	-0.00325,	-0.000969
% body fat	-0.00334***	-0.00498,	-0.00171	-0.00609***	-0.00862,	-0.00355	-0.00575**	-0.00828,	-0.00322	-0.00516*	-0.00761,	-0.0027
**Metabolic Syndrome**	-0.0734***	-0.0931,	-0.0538	-0.0153	-0.0317,	0.0011	-0.013	-0.0284,	0.00238	-0.00985	-0.0244,	0.00469

The sample size for components that used the fasting weights was 3,407 (LDL cholesterol, triglycerides, glucose, and insulin resistance); sample size for all other measures was 7,252. The dependent variable in all models is LTL (in kbp). Each line shows coefficients and 95% CIs from separate statistical models. Model 1 does not adjust for any covariables. Model 2 adjusts for the following covariables: race/ethnicity (white, Mexican American, black, and other), gender, foreign birthplace, education (less than high school, high school diploma, more than high school), class of work (white collar high, blue collar high, white collar low, blue collar low, no work), income, marital status (married or living with partner), age (as continuous), and age-squared. Model 3 additionally controls for ever smoked, current smoker, moderate physical activity, and vigorous physical activity. Model 4 additionally includes white blood cells (SI), lymphocytes (%), monocytes (%), basophils (%), eosinophils (%), and neutrophils (%).

*p < 0.05

**p < 0.01

***p < 0.001.

We also examined the full model 4 in a restricted population of individuals aged 25 to 84 who did not have kidney disease, hypertension, diabetes, or cardiovascular disease (S3 Table). Coefficients of association were similar between the full population and the restricted population with the exception of cystatin C, for which coefficient estimates did not have overlapping 95% CIs. In the full population the association with LTL was -0.0391 (95% CI -0.0772–-0.00701), whereas in the restricted healthy population the association with LTL was stronger, -0.24 (95% CI -0.39–-0.904).

We note that only a small number of interactions were observed based on our a priori specification to test for heterogeneity of association based on seven characteristics: female, black, Mexican American, less than a high school education, poverty income ratio, and ages 20 to 44 and ages 65 to 84. Based on these seven factors with 17 cardiovascular biomarkers, we tested 119 interactions (S4 Table). We note interactions for which the null of 0 for the interaction coefficient was not included in the estimated 95% CI. For triglycerides, there was a stronger association for women (interaction coefficient 0.000485; 95% CI 0.0000731–0.000897); for LDL cholesterol, there was a stronger relationship among black participants (interaction coefficient = 0.0020; 95% CI 0.000287–0.00376); for glomerular filtration rate, there was a stronger relationship among black participants (interaction coefficient = 0.000951, 95% CI 0.0000755–0.00183); for percentage of body fat, there was a stronger relationship among black participants (interaction coefficient = 0.00697, 95% CI 0.00311–0.0108); for triglycerides, there was a stronger relationship among Mexican Americans (interaction coefficient = 0.00624, 95% CI 0.0000165–0.00123); for HbA1c, there was a stronger relationship among those with less than a high school diploma (interaction coefficient = 0.042, 95% CI 0.0158–0.0683); for percentage of body fat, there was a weaker relationship among those with lower levels of income (interaction coefficient = -0.00148, 95% CI -0.00232–-0.000643); for diastolic blood pressure, there was a weaker relationship among participants aged 25–44 (interaction coefficient = -0.00288, 95% CI -0.0051–-0.000652); and for glomerular filtration rate, there was a stronger relationship among participants aged 65 and above (interaction coefficient = 0.00107, 95% CI 0.0000132–0.00212). We found a total of nine interactions for which CIs did not include the null of no association, but, as we tested 119 interactions, we would expect six by chance alone. We therefore do not find substantial evidence for there being any systematic differences in associations in different groups of the population, but there is some evidence that there may be stronger associations between LTL and cardiovascular risk biomarkers among black participants.

We also repeated the primary analyses using dichotomous risk indicators of biomarkers, and results were generally similar (Table 4). Most of the associations with continuous measures were replicated in terms of magnitude, even though coefficients differed. Although associations with clinical thresholds of lipids were notably weaker than with the continuous measures, CIs for estimates were generally overlapping. The primary difference was that there was a stronger association with diastolic blood pressure and insulin resistance when using the clinical cut-point that was not observed for the continuous measure.

**Table 4:** Regression of LTL on clinical cut-points of biomarkers, ages 20–84, NHANES 1999–2002.

	Model 1—unadjusted			Model 2—demographic adjusted			Model 3—demographic + clinical adjusted			Model 4—demographic + clinical + cell type adjusted		
	coef	95% CI		coef	95% CI		coef	95% CI		coef	95% CI	
**Lipoproteins**												
HDL cholesterol	-0.035	-0.0772,	0.00711	-0.043*	-0.0784,	-0.0076	-0.0388*	-0.0736,	-0.00393	-0.0311	-0.0655,	0.00339
LDL cholesterol	0.0131	-0.0641,	0.0903	0.0638	-0.0087,	0.136	0.0594	-0.014,	0.133	0.0581	-0.0156,	0.132
Triglycerides	-0.138***	-0.199,	-0.0762	-0.062*	-0.118,	-0.00628	-0.0576*	-0.111,	-0.00422	-0.0504	-0.102,	0.00152
**Blood sugar**												
Glucose	-0.171***	-0.222,	-0.12	0.012	-0.0339,	0.058	0.0154	-0.0291,	0.0598	0.0228	-0.02,	0.0655
Insulin resistance	-0.138***	-0.166,	-0.111	-0.055***	-0.0807,	-0.0292	-0.0527**	-0.0783,	-0.0271	-0.0412*	-0.0685,	-0.0139
HbA1c	-0.175***	-0.212,	-0.138	-0.012	-0.0581,	0.0342	-0.00734	-0.0546,	0.04	0.00645	-0.0423,	0.0552
**Circulatory pressure**												
Systolic blood pressure	-0.22***	-0.27,	-0.17	0.0442*	0.00103,	0.0873	0.0449*	0.00149,	0.0884	0.0455*	0.00137,	0.0897
Diastolic blood pressure	-0.0872**	-0.141,	-0.0331	-0.067*	-0.125,	-0.00918	-0.066*	-0.123,	-0.00858	-0.0674*	-0.126,	-0.00889
Pulse rate	0.0123	-0.0249,	0.0495	-0.0307	-0.0641,	0.0028	-0.0258	-0.058,	0.00649	-0.0161	-0.0482,	0.0161
**Immune function**												
C-reactive protein	-0.189*	-0.363,	-0.015	-0.13	-0.264,	0.00361	-0.119	-0.254,	0.0155	-0.0931	-0.225,	0.0391
Fibrinogin	-0.185***	-0.235,	-0.135	-0.0257	-0.0811,	0.0297	-0.0225	-0.0762,	0.0312	-0.00777	-0.0575,	0.0419
**Kidney function**												
Cystatin C	-0.352***	-0.421,	-0.283	-0.0345	-0.0989,	0.0298	-0.0334	-0.0965,	0.0297	-0.0279	-0.0909,	0.0351
Glomerular filtration rate	-0.386***	-0.506,	-0.267	-0.0744	-0.167,	0.0177	-0.0695	-0.16,	0.021	-0.0608	-0.155,	0.033
Albumin: Creatinine	-0.158***	-0.223,	-0.0924	-0.0312	-0.0912,	0.0289	-0.0285	-0.0884,	0.0313	-0.0235	-0.0819,	0.0349
**Adiposity**												
BMI	-0.0838***	-0.118,	-0.0498	-0.0617**	-0.0936,	-0.0298	-0.0616**	-0.0937,	-0.0294	-0.0526*	-0.0827,	-0.0225
waist circumference	-0.129***	-0.167,	-0.0921	-0.0635**	-0.0987,	-0.0283	-0.0617**	-0.0962,	-0.0271	-0.0509*	-0.0843,	-0.0175
% body fat	-0.188***	-0.231,	-0.145	-0.0489*	-0.0975,	-0.000342	-0.0437	-0.0921,	0.00468	-0.0357	-0.0848,	0.0134
**Metabolic syndrome**	-0.0734***	-0.0931,	-0.0538	-0.0153	-0.0317,	0.0011	-0.013	-0.0284,	0.00238	-0.00985	-0.0244,	0.00469

The sample size for components that used the fasting weights was 3,407 (LDL cholesterol, triglycerides, glucose, and insulin resistance); sample size for all other measures was 7,252. Model 4 adjusts for the following covariables: race/ethnicity (white, Mexican American, black, and other), gender, foreign birthplace, education (less than high school, high school diploma, more than high school), class of work (white collar high, blue collar high, white collar low, blue collar low, no work), income, marital status (married or living with partner), age (as continuous), age-squared, white blood cells (SI), lymphocytes (%), monocytes (%), basophils (%), eosinophils (%), neutrophils (%), ever smoked, current smoker, moderate physical activity, and vigorous physical activity.

*p < 0.05

**p < 0.01

***p < 0.001.

Table 5 considers a model that includes all of the biomarkers in the same multivariable model so as to examine whether predictors remain associated independently of other cardiovascular disease biomarkers. Although interpretation of the coefficients should be done with caution because they are conditional on other biomarkers and the causal ordering of these biomarkers is not well characterized, this model provides an additional perspective on which cardiovascular biomarkers are the strongest relative predictors of LTL. In the model that includes no demographic controls, triglycerides, systolic blood pressure, diastolic blood pressure, cystatin C, glomerular filtration rate, BMI, waist circumference, and body fat percentage are associated with LTL. After controlling for demographic factors, only the coefficient for the association of pulse rate and LTL has estimated 95% CIs that do not include the null of no association. However, these results should be interpreted in conjunction with the other findings, as domains that have more correlated measures, for example measures of adiposity, will be more attenuated by controlling for all of these measures in a single model.

**Table 5:** Multivariable regression of LTL on all biomarkers, age 20–84, NHANES 1999–2002.

	Model 1—unadjusted			Model 2—demographic adjusted		
	coef	95% CI		coef	95% CI	
**Lipoproteins**						
HDL cholesterol	-0.0284	-0.0676,	0.0108	0.0158	-0.0224,	0.054
LDL cholesterol	-0.00285	-0.024,	0.0183	0.00938	-0.00903,	0.0278
Triglycerides	-0.0505*	-0.0902,	-0.0107	-0.0194	-0.055,	0.0163
**Blood sugar**						
Glucose	-0.013	-0.0721,	0.0462	0.0271	-0.0305,	0.0846
Insulin resistance	0.0414*	-0.0142,	0.097	0.0101	-0.039,	0.0593
HbA1c	-0.0277	-0.0913,	0.0359	-0.0172	-0.0796,	0.0452
**Circulatory pressure**						
Systolic blood pressure	-0.0789*	-0.119,	-0.0385	0.00514	-0.0291,	0.0394
Diastolic blood pressure	0.0336*	0.00886,	0.0584	-0.0143	-0.0417,	0.013
Pulse rate	-0.015	-0.0384,	0.00827	-0.0314	-0.0557,	-0.00706
**Proinflammatory**						
C-reactive protein	-0.0138	-0.0663,	0.0387	-0.0286	-0.0781,	0.021
Fibrinogin	-0.0158	-0.0627,	0.031	0.015	-0.0313,	0.0612
**Kidney function**						
Cystatin C	-0.0292**	-0.0483,	-0.0101	-0.0136	-0.0377,	0.0105
Glomerular filtration rate	0.123	0.0858,	0.16	-0.00206	-0.0465,	0.0424
Albumin: Creatinine	0.0434	-0.0159,	0.103	0.0119	-0.0451,	0.069
**Adiposity**						
BMI	0.0705	0.00938,	0.132	-0.0417	-0.108,	0.0241
waist circumference	-0.0809*	-0.147,	-0.0147	0.0306	-0.0359,	0.0971
% body fat	-0.0415	-0.0763,	-0.0067	-0.00753	-0.0477,	0.0327

The effective sample size for each of the models was 3,407 because of the use of fasting sample weights. Model 1 includes only the 17 biomarkers shown in the table. Model 2 additionally adjusts for the following covariables: race/ethnicity (white, Mexican American, black, and other), gender, foreign birthplace, education (less than high school, high school diploma, more than high school), class of work (white collar high, blue collar high, white collar low, blue collar low, no work), income, marital status (married or living with partner), age (as continuous), and age-squared.

**Fig 5:**
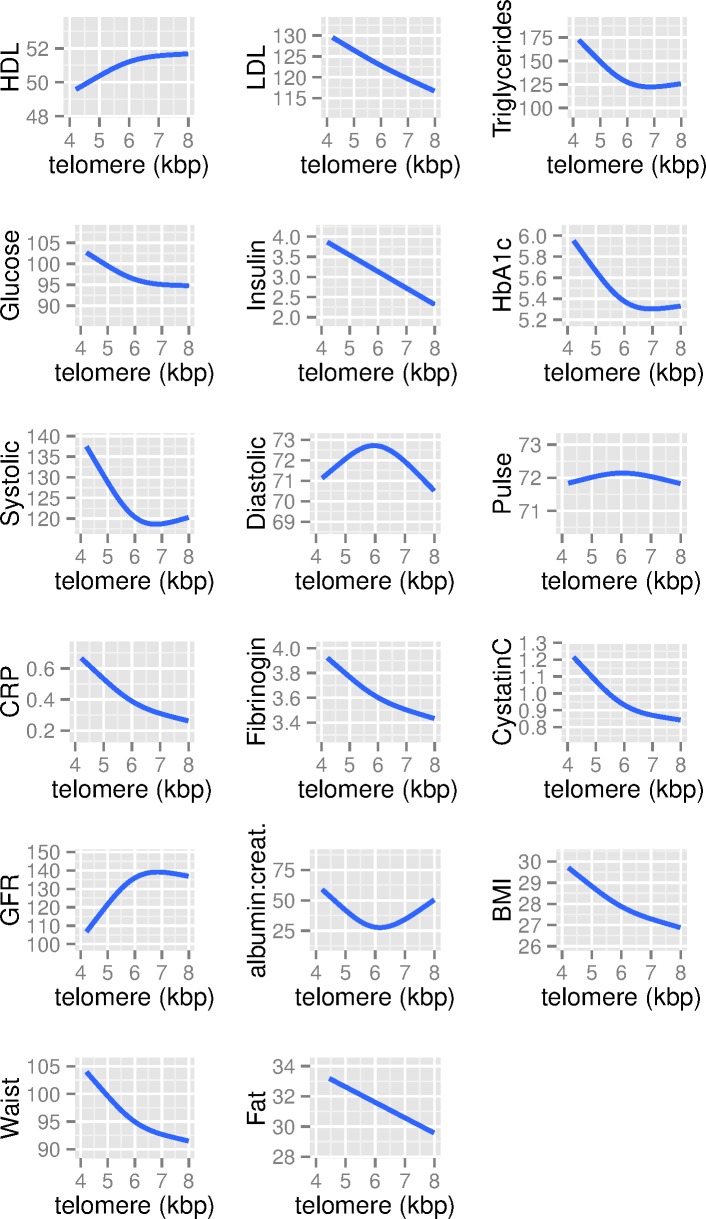
Generalized additive mixed models of the association between biomarkers and LTL (in kb pairs), ages 20–84, NHANES 1999–2002.

Finally, we also examine the potential for nonlinear relationships between levels of cardiovascular biomarkers and LTL (Fig 5). In general, relationships do not deviate strongly from linearity, albeit with a few exceptions. With glucose, HbA1c, glomerular filtration rate, and systolic blood pressure, there is a weakening of the association above 6.5 kb pairs. Thus, even though there is a nonlinear relationship, there tends to be a linear association across the bulk of the data (between 5 and 6.5 kb pairs as shown in Fig 3), with nonlinearity existing primarily above 6.5 kb pairs. The exception to this is for diastolic blood pressure, for which there is an inverted U-shaped relationship.

## Discussion

The purpose of our study was to examine whether LTL is independent of other known biological risk markers of cardiovascular disease. This is an important question given associations of LTL with cardiovascular disease mortality. The significance of this question is whether across the life course LTL is independent of already well-documented biological mechanisms of cardiovascular disease risk. Our findings suggest that, among a nationally representative US population, there are moderate correlations between LTL and important cardiovascular risk factors, even after accounting for age, social and demographic factors, health behaviors, and white blood cell types. We found that controlling for cell type reduced magnitudes of association by between 10% and 20%.

We found association with LTL for two measure of lipoprotein (HDL cholesterol, triglycerides), one measure of blood sugar (insulin resistance), three measures of circulatory pressure (systolic blood pressure, diastolic blood pressure, and pulse rate), one proinflammatory marker (C-reactive protein), one measure of kidney function (cystatin C), and all three measures of adiposity (BMI, waist circumference, percentage of body fat). Among adiposity measures, percentage of body fat had the strongest association in independent models. LTL was not associated with a measure of overall cardiovascular risk—the metabolic syndrome—because of its unique association with particular cardiovascular biomarkers. This study also allowed us to examine whether these relationships differed by age, gender, race/ethnicity, education, and income. This question is important because relationships between LTL and mortality have been found to differ by some of these domains [45]. In examining interactions between LTL and the 17 biomarkers, we found only slightly more interactions than would be expected by chance alone, suggesting that the relationships we present are fairly consistent across demographic groups.

The strengths of this study are that we for the first time examine telomere length with biomarkers that are risk factors for morbidity and mortality in a multi-ethnic nationally representative sample of the US population. We also examined these associations across a broad age range of the population, from 25 to 84, based on interest in how LTL acts as a marker of biological aging across the life course. In addition, we examine this in a general population sample controlling for a greater number of potentially confounding factors as compared to other studies. We also are able to examine a wide range of cardiovascular risk biomarkers given the detailed measurements available in the NHANES data. No studies we are aware of have been able to examine this many biomarkers in a comparative manner nor to control for white cell type frequencies. Our findings should be considered generalizable to the US noninstitutionalized population from the ages of 25 to 84.

The study has several limitations. Most importantly, our results do not contribute to an understanding of the directionality of any associations because of the cross-sectional nature of the data. Causal inference from our findings is not possible. This refers to both the direction of potential causation between cardiovascular risk markers and LTL as well as the potential confounding of the biomarkers themselves. For example, it may be a dietary factor, rather than adiposity, that is the direct cause of changes in LTL. However, the goal of our study is to determine whether LTL is largely independent of other cardiovascular disease risk markers, and, thus, causal inference is not our intention. Randomized trials and quasi-experimental methods are more suited to the separate and also important question of how LTL may act as a cause or marker for cardiovascular disease risk. Secondly, although not a limitation per se, our results must be interpreted specifically with respect to LTL, because telomere length is cell type specific. Moreover, although we found that controlling for broad measures of cell type reduced magnitudes of association, we were not able to examine specific types of T and B cells where different telomere lengths have been demonstrated [15]. All of our conclusions about which physiological systems LTL is related to are limited by the commonly used clinical measures of those systems. In addition, we were not able to perform a mediation analysis for cardiovascular mortality due to the weak relationship between LTL and relatively short-term (mean 9.5 years) cardiovascular mortality follow-up available from NHANES [45]. In addition, although we use standard clinical cut-points for our analysis, we do not take into account that these may differ for best differentiating risk in population subgroups. Finally, our baseline data come from 1999–2002 and not from more recent data collection. Several facts mitigate this potential limitation. First, the actual telomere length assays were done in 2011, using more recent methodological approaches, and, thus, the assays themselves were done recently on stored DNA. Secondly, most of the population aged 25–84 that were part of the collection of data in 1999–2002 are still alive, so inference is still about the current US population. We are also not aware of any data suggesting that the relationship between LTL and cardiovascular biomarkers has changed since 1999–2002.

The results from our study can be compared to other studies that have previously examined similar factors and LTL. As mentioned in the introduction, the largest prior analysis found similar findings with respect to adiposity related measures and inflammation [8], with our study confirming these findings in a larger multi-ethnic US population. The most commonly examined cardiovascular risk factor with LTL has been BMI, and a recent meta-analysis summarized the results of five prior studies that controlled for covariables in regression analyses, finding an overall adjusted regression coefficient of -0.008 kBP per BMI unit [46] from 4,183 individuals. Our single study of a larger nationally representative population found a weaker association of -0.0047 kBP per BMI unit, which may in part be due to our ability to control for a greater number of potentially confounding factors.

We also found inverse associations with an inflammatory marker (C-reactive protein) in contrast with prior, more limited studies. A prior study in a sample of individuals over the age of 70 did not find an association with C-reactive protein [47]. The differences in our findings are, however, consistent with genetic instrumental variables findings showing SNPs for C-reactive protein are associated with LTL [48]. In comparison to prior work [49], we did not find associations with measures of blood sugar and systolic blood pressure. Our findings for systolic blood pressure were also different from findings in a Costa Rican population, in which higher systolic blood pressure was found to be associated with longer telomere length [9].

Our findings can also be compared to those from an environmental wide association study between LTL and 461 environmental, behavioral, and clinical variables using the same NHANES data [50]. This study found 22 out of 461 associations, with false discovery rate of <5%. Comparable findings similar to those in our study include C-reactive protein, trunk fat (a measure of adiposity), and pulse rate. Our approach of targeting 17 cardiovascular disease biomarkers with a hypothesis-driven approach gave us more power to detect associations than in this prior study, which is the most likely explanation for our findings with additional adiposity measures, diastolic blood pressure, and HDL cholesterol. In addition, we controlled for white blood cell type, examined clinical cut-points of biomarkers, and examined nonlinearity of associations. As shown in our study, we missed the association between LTL and diastolic blood pressure when using a continuous measure rather than a clinical cut-point. Our findings were also similar to this prior study in finding little evidence for effect measure modification by gender and race/ethnicity.

Our study has implications for understanding how LTL may be relevant as a biomarker of aging. LTL is most associated with adiposity, immune function, diastolic blood pressure, pulse, and HDL cholesterol. This gives suggestions to the underlying role for LTL as a marker for coronary heart disease risk through oxidative stress-related pathways resulting in immune cell turnover and subsequent shorter LTL [47], consistent with the links between inflammation, adiposity, and oxidative stress [51]. Furthermore, our evidence is consistent with LTL as related to different aspects of biological dysregulation. Given the range of biomarkers measured in this large sample, it enabled us to test whether LTL truly reflects cumulative dysregulation across systems. The findings suggest that telomere length is indeed related to biomarkers of multiple regulatory systems that indicate risk for cardiovascular disease and possibly other diseases as well. There were relations across adiposity, inflammatory markers, and circulatory pressure. Multiple dysregulation across systems has been measured as a summary variable of unhealthy values across systems called allostatic load. Here, it appears LTL may serve as a cellular-based indicator of systemic allostatic load.

However, there were not relations across all systems; specifically, there were weaker or nonexistent relationships with blood sugar. With respect to this finding, prior studies have found differences in LTL between those with and without diabetes [52], which may be a more extreme comparison than looking at whether there is an association in a representative sample of the population. We do find consistent results in our basic model that does not control for demographic confounding variables, but this association is eliminated after controlling for a large set of potential confounding factors, which may also explain differences in results. A meta-analysis identifying an association between LTL and diabetes did not examine what potential confounding factors were controlled for [53]. In addition, studies showing greater mortality for those with shorter telomere length within diabetic populations [54] are more informative about the role of telomere length in the progression of disease rather than in the general population. With respect to kidney function, many prior studies reporting an association with LTL were in clinical samples, not in the general population [55]. Other work showing an association with kidney function and aging has been using telomere length from kidney tissue, not leukocytes [56]. Thus, although blood sugar and kidney function clearly contribute to cardiovascular risk, their dysregulation in the general population does not seem to be captured by LTL, with the exception of some of our models showing a weak association with cystatin C. Again, however, it is important to consider that we used only established clinical risk factors for cardiovascular disease to capture the association of LTL with different physiological systems. Work taking a metabolomics approach has identified more specific biological components associated with LTL [57].

Future studies should continue to investigate the relationship between known cardiovascular biomarkers and LTL in more dynamic ways, including changes over time in both biomarkers and LTL. Most critically, in order to understand the clinical relevance for cardiovascular disease, longer duration follow-up studies that allow for mediation analysis with clinical endpoints must be designed to identify where there may be value added for the assay of LTL beyond traditional cardiovascular biomarkers. Finally, more complete measures of proinflammatory response should be examined to more specifically investigate how LTL is a marker of this system, ideally on cells that have been sorted by type [15].

## Supporting Information

S1 STROBE Checklist (DOC)

S1 Table Association of LTL with concentration of white blood cells and constituent cell types, ages 20 to 84, population without chronic disease, NHANES 1999–2002. (DOCX)

S2 Table Interaction terms for association of LTL with concentration of white blood cells and constituent cell types, ages 20 to 84, NHANES 1999–2002. (DOCX)

S3 Table Regression of LTL on continuous biomarkers, ages 20 to 84, population without chronic disease, NHANES 1999–2002. (DOCX)

S4 Table Interaction terms for association of LTL on continuous biomarkers, ages 20 to 84, NHANES 1999–2002. (DOCX)

S5 Table Multivariable regression of LTL on all biomarkers, ages 20–84, population without chronic disease, NHANES 1999–2002. (DOCX)

## Abbreviations

CDC: US Centers for Disease Control and Prevention
HDL: high-density lipoprotein
LDL: low-density lipoprotein
LTL: leukocyte telomere length
NHANES: National Health and Nutrition Examination Survey
SNP: single nucleotide polymorphism
