# Supplementary material for: Leukocyte Telomere Length in Relation to 17 Biomarkers of Cardiovascular Disease Risk: A Cross-Sectional Study of US Adults
Source: PLoS Med. 2016 Nov 29;13(11):e1002188. doi: 10.1371/journal.pmed.1002188 (PMC5127504; doi:10.1371/journal.pmed.1002188)
Supplement: S1 Table — (DOCX) [file pmed.1002188.s002.docx]

|  | **Model 3 – demographic + health related behaviors** | | |
| --- | --- | --- | --- |
|  | **coef** | **95% CI** | |
| White blood cells (SI) | -0.0148 | -0.0266, | -0.00297 |
| Lymphocytes (%) | 0.00134 | -0.00112, | 0.0038 |
| Monocytes (%) | 0.000637 | -0.00899, | 0.0103 |
| Neutrophils (%) | -0.00111 | -0.00338, | 0.00115 |
| Eosinophils (%) | -0.00149 | -0.00809, | 0.00512 |
| Basophils (%) | 0.0519 | 0.0016, | 0.102 |

Sample size for all models was 4772. The dependent variable in all models is leukocyte telomere length (in kbp). Model 3 adjusts for the following covariates: race/ethnicity (white, Mexican American, black and other), gender, foreign birthplace, education (less than high school, high school diploma, more than high school), class of work (White collar high, Blue collar high, White collar low, Blue collar low, no work), income, marital status (married or living with partner), age (as continuous), age-squared, ever smoked, current smoker, moderate physical activity and vigorous physical activity.
