# Supplementary material for: Leukocyte Telomere Length in Relation to 17 Biomarkers of Cardiovascular Disease Risk: A Cross-Sectional Study of US Adults
Source: PLoS Med. 2016 Nov 29;13(11):e1002188. doi: 10.1371/journal.pmed.1002188 (PMC5127504; doi:10.1371/journal.pmed.1002188)
Supplement: S2 Table — (DOCX) [file pmed.1002188.s003.docx]

| **Interaction with Female** | |  |  |
| --- | --- | --- | --- |
|  | **Model 3 – demographic + health related behaviors** | | |
|  | **coef** | **95% CI** | |
| White blood cells (SI) | -0.00211 | -0.0112, | 0.00697 |
| Lymphocytes (%) | -0.00213 | -0.00553, | 0.00128 |
| Monocytes (%) | -0.01 | -0.0228, | 0.0028 |
| Neutrophils (%) | 0.00245 | -0.00036, | 0.00525 |
| Eosinophils (%) | -0.00376 | -0.0153, | 0.0078 |
| Basophils (%) | -0.023 | -0.0932, | 0.0472 |
|  |  |  |  |
| **Interaction with Black** | |  |  |
|  | **Model 3 – demographic + health related behaviors** | | |
|  | **coef** | **95% CI** | |
| White blood cells (SI) | 0.00636 | -0.00924, | 0.022 |
| Lymphocytes (%) | 0.00405 | -0.000993, | 0.0091 |
| Monocytes (%) | -0.0202 | -0.0336, | -0.00684 |
| Neutrophils (%) | -0.00231 | -0.00678, | 0.00215 |
| Eosinophils (%) | 0.00525 | -0.00526, | 0.0158 |
| Basophils (%) | -0.048 | -0.115, | 0.0193 |
|  |  |  |  |
| **Interaction with Mexican American** | | |  |
|  | **Model 3 – demographic + health related behaviors** | | |
|  | **coef** | **95% CI** | |
| White blood cells (SI) | 0.00545 | -0.0151, | 0.026 |
| Lymphocytes (%) | -0.00178 | -0.00674, | 0.00319 |
| Monocytes (%) | 0.00353 | -0.0113, | 0.0184 |
| Neutrophils (%) | 0.00076 | -0.00332, | 0.00484 |
| Eosinophils (%) | 0.00759 | -0.00965, | 0.0248 |
| Basophils (%) | -0.0347 | -0.128, | 0.0587 |
|  |  |  |  |
| **Interaction with less than a high school diploma** | | | |
|  | **Model 3 – demographic + health related behaviors** | | |
|  | **coef** | **95% CI** | |
| White blood cells (SI) | 0.000349 | -0.0196, | 0.0203 |
| Lymphocytes (%) | 0.00021 | -0.0053, | 0.00573 |
| Monocytes (%) | -0.00251 | -0.0156, | 0.0106 |
| Neutrophils (%) | 0.00023 | -0.00403, | 0.00449 |
| Eosinophils (%) | -0.00095 | -0.0126, | 0.0107 |
| Basophils (%) | 0.0151 | -0.0753, | 0.105 |
|  |  |  |  |
| **Interaction with income** | |  |  |
|  | **Model 3 – demographic + health related behaviors** | | |
|  | **coef** | **95% CI** | |
| White blood cells (SI) | -0.000881 | -0.00521, | 0.00345 |
| Lymphocytes (%) | 0.000752 | -0.000469, | 0.00197 |
| Monocytes (%) | -0.0000137 | -0.00342, | 0.00339 |
| Neutrophils (%) | -0.00096 | -0.00201, | 0.0000895 |
| Eosinophils (%) | 0.00469 | 0.00149, | 0.00789 |
| Basophils (%) | 0.00763 | -0.0122, | 0.0275 |
|  |  |  |  |
| **Interaction with age 25-44** | |  |  |
|  | **Model 3 – demographic + health related behaviors** | | |
|  | **coef** | **95% CI** | |
| White blood cells (SI) | -0.00579 | -0.0182, | 0.00664 |
| Lymphocytes (%) | 0.00158 | -0.0021, | 0.00526 |
| Monocytes (%) | 0.00538 | -0.00832, | 0.0191 |
| Neutrophils (%) | -0.00144 | -0.00482, | 0.00194 |
| Eosinophils (%) | -0.00116 | -0.0171, | 0.0148 |
| Basophils (%) | -0.0447 | -0.11, | 0.0202 |
|  |  |  |  |
| **Interaction with age 65 and above** | | |  |
|  | **Model 3 – demographic + health related behaviors** | | |
|  | **coef** | **95% CI** | |
| White blood cells (SI) | -0.00625 | -0.0199, | 0.00743 |
| Lymphocytes (%) | -0.00348 | -0.00657, | -0.000391 |
| Monocytes (%) | -0.00327 | -0.0185, | 0.012 |
| Neutrophils (%) | 0.00290 | 0.000418, | 0.00539 |
| Eosinophils (%) | 0.00283 | -0.009, | 0.0147 |
| Basophils (%) | 0.0183 | -0.0591, | 0.0956 |
