# Supplementary material for: Leukocyte Telomere Length in Relation to 17 Biomarkers of Cardiovascular Disease Risk: A Cross-Sectional Study of US Adults
Source: PLoS Med. 2016 Nov 29;13(11):e1002188. doi: 10.1371/journal.pmed.1002188 (PMC5127504; doi:10.1371/journal.pmed.1002188)
Supplement: S3 Table — (DOCX) [file pmed.1002188.s004.docx]

|  | **Model 4 - demographic + health related behaviors + cell type adjusted** | | |
| --- | --- | --- | --- |
|  | **coef** | **95% CI** | |
| Lipoproteins |  |  |  |
| HDL cholesterol | 0.00237 | 0.000701, | 0.00404 |
| LDL cholesterol | -0.000109 | -0.00115, | 0.00093 |
| Triglycerides | -0.000398 | -0.000834, | 0.0000376 |
| Blood sugar |  |  |  |
| Glucose | 0.00106 | 0.00007, | 0.00205 |
| Insulin resistance | 0.000864 | -0.0105, | 0.0122 |
| HbA1c | -0.00711 | -0.0495, | 0.0353 |
| Circulatory pressure |  |  |  |
| Systolic blood pressure | 0.000483 | -0.000791, | 0.00176 |
| Diastolic blood pressure | -0.00108 | -0.00279, | 0.000635 |
| Pulse rate | -0.00139 | -0.00295, | 0.000177 |
| Immune function |  |  |  |
| C-reactive protein | -0.0365 | -0.0697, | -0.00331 |
| Fibrinogin | -0.0164 | -0.0676, | 0.0347 |
| Kidney function |  |  |  |
| Cystatin C | -0.24 | -0.39, | -0.0904 |
| Glomelular filtration rate | 0.000172 | -0.00058, | 0.000925 |
| Albumin : Creatinine | -0.000102 | -0.000256, | 0.0000511 |
| Adiposity |  |  |  |
| BMI | -0.00566 | -0.00931, | -0.00201 |
| waist circumference | -0.00252 | -0.0039, | -0.00114 |
| % body fat | -0.00419 | -0.00717, | -0.00121 |
| Metabolic syndrome | -0.0104 | -0.0299, | 0.00922 |

The sample size for components that used the fasting weights was 2190 (LDL cholesterol, Triglycerides, Glucose and Insulin resistance), sample size for all other measures was 4772. Model 4 adjusts for the following covariates: race/ethnicity (white, Mexican American, black and other), gender, foreign birthplace, education (less than high school, high school diploma, more than high school), class of work (White collar high, Blue collar high, White collar low, Blue collar low, no work), income, marital status (married or living with partner), age (as continuous), age-squared, white blood cells (SI), lymphocytes (%), monocytes (%), basophils (%), eosinophils (%), neutrophils (%), ever smoked, current smoker, moderate physical activity and vigorous physical activity.
