# Supplementary material for: Leukocyte Telomere Length in Relation to 17 Biomarkers of Cardiovascular Disease Risk: A Cross-Sectional Study of US Adults
Source: PLoS Med. 2016 Nov 29;13(11):e1002188. doi: 10.1371/journal.pmed.1002188 (PMC5127504; doi:10.1371/journal.pmed.1002188)
Supplement: S4 Table — (DOCX) [file pmed.1002188.s005.docx]

| **Interaction terms with Female** | | | | |  | | | |  | | | |  |  |  |
| --- | --- | --- | --- | --- | --- | --- | --- | --- | --- | --- | --- | --- | --- | --- | --- |
|  | **Model 4 - demographic + health related behaviors + cell type adjusted** | | | | | | | | | | | |  |  |  |
|  | **coef** | | | | **95% CI** | | | | | | | |  |  |  |
| Lipoproteins | | |  | | | |  | | | |  | | | |  |
| HDL cholesterol | | | -0.000922 | | | | -0.00318, | | | | 0.00133 | | | |  |
| LDL cholesterol | | | -0.000172 | | | | -0.00154, | | | | 0.0012 | | | |  |
| Triglycerides | | | 0.000485 | | | | 0.0000731, | | | | 0.000897 | | | |  |
| Blood sugar | | |  | | | |  | | | |  | | | |  |
| Glucose | | | 0.00129 | | | | -0.000244, | | | | 0.00282 | | | |  |
| Insulin resistance | | | 0.0097 | | | | -0.00354, | | | | 0.0229 | | | |  |
| HbA1c | | | 0.0141 | | | | -0.0155, | | | | 0.0437 | | | |  |
| Circulatory pressure | | |  | | | |  | | | |  | | | |  |
| Systolic blood pressure | | | -0.000345 | | | | -0.00175, | | | | 0.00106 | | | |  |
| Diastolic blood pressure | | | -0.000241 | | | | -0.00285, | | | | 0.00236 | | | |  |
| Pulse rate | | | -0.000131 | | | | -0.00172, | | | | 0.00146 | | | |  |
| Immune function | | |  | | | |  | | | |  | | | |  |
| C-reactive protein | | | 0.0335 | | | | -0.0056, | | | | 0.0725 | | | |  |
| Fibrinogin | | | 0.0207 | | | | -0.0265, | | | | 0.0679 | | | |  |
| Kidney function | | |  | | | |  | | | |  | | | |  |
| Cystatin C | | | 0.0253 | | | | -0.0496, | | | | 0.1 | | | |  |
| Glomelular filtration rate | | | -0.0000732 | | | | -0.00123, | | | | 0.00109 | | | |  |
| Albumin : Creatinine | | | 0.00000298 | | | | -0.00014, | | | | 0.000146 | | | |  |
| Adiposity | | |  | | | |  | | | |  | | | |  |
| BMI | | | -0.000512 | | | | -0.00415, | | | | 0.00313 | | | |  |
| waist circumference | | | 0.0000765 | | | | -0.00138, | | | | 0.00154 | | | |  |
| % body fat | | | 0.000768 | | | | -0.00469, | | | | 0.00623 | | | |  |
| Metabolic syndrome | | | 0.0301 | | | | -0.00735, | | | | 0.0676 | | | |  |
|  |  | | | |  | | | |  | | | |  |  |  |
| **Interactions with Black** |  | | | |  | | | |  | | | |  |  |  |
|  | **Model 4 - demographic + health related behaviors + cell type adjusted** | | | | | | | | | | | |  |  |  |
|  | **coef** | | | | **95% CI** | | | | | | | |  |  |  |
| Lipoproteins | | | |  | | | |  | | | |  | | | |
| HDL cholesterol | | | | -0.00173 | | | | -0.00405, | | | | 0.000585 | | | |
| LDL cholesterol | | | | 0.00202 | | | | 0.000287, | | | | 0.00376 | | | |
| Triglycerides | | | | -0.000164 | | | | -0.00124, | | | | 0.000913 | | | |
| Blood sugar | | | |  | | | |  | | | |  | | | |
| Glucose | | | | 0.000511 | | | | -0.000988, | | | | 0.00201 | | | |
| Insulin resistance | | | | -0.01 | | | | -0.0223, | | | | 0.00226 | | | |
| HbA1c | | | | 0.0198 | | | | -0.0162, | | | | 0.0558 | | | |
| Circulatory pressure | | | |  | | | |  | | | |  | | | |
| Systolic blood pressure | | | | -0.00104 | | | | -0.00267, | | | | 0.000584 | | | |
| Diastolic blood pressure | | | | 0.000478 | | | | -0.00201, | | | | 0.00297 | | | |
| Pulse rate | | | | 0.00133 | | | | -0.00141, | | | | 0.00406 | | | |
| Immune function | | | |  | | | |  | | | |  | | | |
| C-reactive protein | | | | 0.00573 | | | | -0.0327, | | | | 0.0441 | | | |
| Fibrinogin | | | | -0.0134 | | | | -0.0712, | | | | 0.0445 | | | |
| Kidney function | | | |  | | | |  | | | |  | | | |
| Cystatin C | | | | -0.000111 | | | | -0.0524, | | | | 0.0521 | | | |
| Glomelular filtration rate | | | | 0.000951 | | | | 0.0000755, | | | | 0.00183 | | | |
| Albumin : Creatinine | | | | -0.0000652 | | | | -0.000196, | | | | 0.0000654 | | | |
| Adiposity | | | |  | | | |  | | | |  | | | |
| BMI | | | | 0.00346 | | | | -0.00286, | | | | 0.00977 | | | |
| waist circumference | | | | 0.000758 | | | | -0.00171, | | | | 0.00322 | | | |
| % body fat | | | | 0.00697 | | | | 0.00311, | | | | 0.0108 | | | |
| Metabolic syndrome | | | | -0.0152 | | | | -0.0588, | | | | 0.0285 | | | |
|  |  | | | |  | | | |  | | | |  |  |  |
| **Interactions with Mexican American** | | | | |  | | | |  | | | |  |  |  |
|  | **Model 4 - demographic + health related behaviors + cell type adjusted** | | | | | | | | | | | |  |  |  |
|  | **coef** | | | | **95% CI** | | | | | | | |  |  |  |
| Lipoproteins | |  | | | |  | | | |  | | | |  |  |
| HDL cholesterol | | -0.00198 | | | | -0.00421, | | | | 0.000245 | | | |  |  |
| LDL cholesterol | | -0.000505 | | | | -0.00177, | | | | 0.000757 | | | |  |  |
| Triglycerides | | 0.000624 | | | | 0.0000165, | | | | 0.00123 | | | |  |  |
| Blood sugar | |  | | | |  | | | |  | | | |  |  |
| Glucose | | -0.000405 | | | | -0.00178, | | | | 0.000968 | | | |  |  |
| Insulin resistance | | -0.000723 | | | | -0.0143, | | | | 0.0128 | | | |  |  |
| HbA1c | | 0.00774 | | | | -0.0274, | | | | 0.0429 | | | |  |  |
| Circulatory pressure | |  | | | |  | | | |  | | | |  |  |
| Systolic blood pressure | | -0.00116 | | | | -0.00307, | | | | 0.000741 | | | |  |  |
| Diastolic blood pressure | | -0.00238 | | | | -0.00552, | | | | 0.000752 | | | |  |  |
| Pulse rate | | 0.00176 | | | | -0.00131, | | | | 0.00482 | | | |  |  |
| Immune function | |  | | | |  | | | |  | | | |  |  |
| C-reactive protein | | 0.0248 | | | | -0.00506, | | | | 0.0547 | | | |  |  |
| Fibrinogin | | 0.0136 | | | | -0.0402, | | | | 0.0674 | | | |  |  |
| Kidney function | |  | | | |  | | | |  | | | |  |  |
| Cystatin C | | 0.1 | | | | -0.000457, | | | | 0.201 | | | |  |  |
| Glomelular filtration rate | | -0.000656 | | | | -0.0015, | | | | 0.000191 | | | |  |  |
| Albumin : Creatinine | | -0.0000525 | | | | -0.000159, | | | | 0.0000541 | | | |  |  |
| Adiposity | |  | | | |  | | | |  | | | |  |  |
| BMI | | 0.000109 | | | | -0.00555, | | | | 0.00577 | | | |  |  |
| waist circumference | | 0.000279 | | | | -0.00203, | | | | 0.00259 | | | |  |  |
| % body fat | | -0.000967 | | | | -0.00427, | | | | 0.00234 | | | |  |  |
| Metabolic syndrome | | 0.0165 | | | | -0.027, | | | | 0.06 | | | |  |  |
|  |  | | | |  | | | |  | | | |  |  |  |
| **Interactions with less than a high school diploma** | | | | |  | | | |  | | | |  |  |  |
|  | **Model 4 - demographic + health related behaviors + cell type adjusted** | | | | | | | | | | | |  |  |  |
|  | **coef** | | | | **95% CI** | | | | | | | |  |  |  |
| Lipoproteins | | | |  | | | |  | | | |  | | | |
| HDL cholesterol | | | | 0.000475 | | | | -0.00211, | | | | 0.00306 | | | |
| LDL cholesterol | | | | -0.000302 | | | | -0.00148, | | | | 0.000877 | | | |
| Triglycerides | | | | 0.000178 | | | | -0.000474, | | | | 0.00083 | | | |
| Blood sugar | | | |  | | | |  | | | |  | | | |
| Glucose | | | | 0.00149 | | | | -0.000141, | | | | 0.00312 | | | |
| Insulin resistance | | | | 0.00902 | | | | -0.00442, | | | | 0.0225 | | | |
| HbA1c | | | | 0.042 | | | | 0.0158, | | | | 0.0683 | | | |
| Circulatory pressure | | | |  | | | |  | | | |  | | | |
| Systolic blood pressure | | | | -0.000465 | | | | -0.00176, | | | | 0.000825 | | | |
| Diastolic blood pressure | | | | 0.00112 | | | | -0.00118, | | | | 0.00342 | | | |
| Pulse rate | | | | 0.000482 | | | | -0.00216, | | | | 0.00312 | | | |
| Immune function | | | |  | | | |  | | | |  | | | |
| C-reactive protein | | | | -0.0119 | | | | -0.0395, | | | | 0.0158 | | | |
| Fibrinogin | | | | 0.0031 | | | | -0.0371, | | | | 0.0433 | | | |
| Kidney function | | | |  | | | |  | | | |  | | | |
| Cystatin C | | | | -0.0146 | | | | -0.083, | | | | 0.0538 | | | |
| Glomelular filtration rate | | | | 0.000283 | | | | -0.000468, | | | | 0.00103 | | | |
| Albumin : Creatinine | | | | -0.0000278 | | | | -0.000125, | | | | 0.0000696 | | | |
| Adiposity | | | |  | | | |  | | | |  | | | |
| BMI | | | | 0.00285 | | | | -0.00181, | | | | 0.0075 | | | |
| waist circumference | | | | 0.000636 | | | | -0.00161, | | | | 0.00288 | | | |
| % body fat | | | | 0.00241 | | | | -0.000856, | | | | 0.00568 | | | |
| Metabolic syndrome | | | | 0.0197 | | | | -0.0179, | | | | 0.0574 | | | |
|  |  | | | |  | | | |  | | | |  |  |  |
| **Interactions with income** |  | | | |  | | | |  | | | |  |  |  |
|  | **Model 4 - demographic + health related behaviors + cell type adjusted** | | | | | | | | | | | |  |  |  |
|  | **coef** | | | | **95% CI** | | | | | | | |  |  |  |
| Lipoproteins | |  | | | |  | | | |  | | | |  |  |
| HDL cholesterol | | -0.000188 | | | | -0.00079, | | | | 0.000414 | | | |  |  |
| LDL cholesterol | | 0.000248 | | | | -0.0001, | | | | 0.000596 | | | |  |  |
| Triglycerides | | 0.0000604 | | | | -0.000122, | | | | 0.000243 | | | |  |  |
| Blood sugar | |  | | | |  | | | |  | | | |  |  |
| Glucose | | -0.000124 | | | | -0.000614, | | | | 0.000366 | | | |  |  |
| Insulin resistance | | -0.000736 | | | | -0.00477, | | | | 0.0033 | | | |  |  |
| HbA1c | | -0.00958 | | | | -0.0222, | | | | 0.00301 | | | |  |  |
| Circulatory pressure | |  | | | |  | | | |  | | | |  |  |
| Systolic blood pressure | | 0.000482 | | | | -0.0000471, | | | | 0.00101 | | | |  |  |
| Diastolic blood pressure | | -0.0000119 | | | | -0.000825, | | | | 0.000801 | | | |  |  |
| Pulse rate | | 0.000164 | | | | -0.000664, | | | | 0.000993 | | | |  |  |
| Immune function | |  | | | |  | | | |  | | | |  |  |
| C-reactive protein | | -0.00106 | | | | -0.0121, | | | | 0.00997 | | | |  |  |
| Fibrinogin | | -0.00862 | | | | -0.0275, | | | | 0.0103 | | | |  |  |
| Kidney function | |  | | | |  | | | |  | | | |  |  |
| Cystatin C | | -0.00452 | | | | -0.0384, | | | | 0.0294 | | | |  |  |
| Glomelular filtration rate | | -0.000107 | | | | -0.000347, | | | | 0.000133 | | | |  |  |
| Albumin : Creatinine | | 0.00000553 | | | | -0.000045, | | | | 0.0000561 | | | |  |  |
| Adiposity | |  | | | |  | | | |  | | | |  |  |
| BMI | | -0.0011 | | | | -0.00267, | | | | 0.000464 | | | |  |  |
| waist circumference | | -0.000195 | | | | -0.000847, | | | | 0.000457 | | | |  |  |
| % body fat | | -0.00148 | | | | -0.00232, | | | | -0.000643 | | | |  |  |
| Metabolic syndrome | | 0.00691 | | | | -0.00517, | | | | 0.019 | | | |  |  |
|  |  | | | |  | | | |  | | | |  |  |  |
| **Interactions with age 25-44** |  | | | |  | | | |  | | | |  |  |  |
|  | **Model 4 - demographic + health related behaviors + cell type adjusted** | | | | | | | | | | | |  |  |  |
|  | **coef** | | | | **95% CI** | | | | | | | |  |  |  |
| Lipoproteins | | |  | | | |  | | | |  | | | |  |
| HDL cholesterol | | | 0.00119 | | | | -0.00068, | | | | 0.00306 | | | |  |
| LDL cholesterol | | | 0.000459 | | | | -0.000728, | | | | 0.00165 | | | |  |
| Triglycerides | | | 0.0000596 | | | | -0.000359, | | | | 0.000479 | | | |  |
| Blood sugar | | |  | | | |  | | | |  | | | |  |
| Glucose | | | -0.000111 | | | | -0.00148, | | | | 0.00126 | | | |  |
| Insulin resistance | | | -0.00456 | | | | -0.0184, | | | | 0.00927 | | | |  |
| HbA1c | | | -0.00993 | | | | -0.0495, | | | | 0.0296 | | | |  |
| Circulatory pressure | | |  | | | |  | | | |  | | | |  |
| Systolic blood pressure | | | -0.00128 | | | | -0.00352, | | | | 0.000969 | | | |  |
| Diastolic blood pressure | | | -0.00288 | | | | -0.0051, | | | | -0.000652 | | | |  |
| Pulse rate | | | -0.00158 | | | | -0.00471, | | | | 0.00155 | | | |  |
| Immune function | | |  | | | |  | | | |  | | | |  |
| C-reactive protein | | | -0.00697 | | | | -0.0468, | | | | 0.0328 | | | |  |
| Fibrinogin | | | -0.0382 | | | | -0.0978, | | | | 0.0214 | | | |  |
| Kidney function | | |  | | | |  | | | |  | | | |  |
| Cystatin C | | | 0.00253 | | | | -0.0862, | | | | 0.0912 | | | |  |
| Glomelular filtration rate | | | -0.000655 | | | | -0.00146, | | | | 0.000147 | | | |  |
| Albumin : Creatinine | | | -0.0000525 | | | | -0.000166, | | | | 0.0000608 | | | |  |
| Adiposity | | |  | | | |  | | | |  | | | |  |
| BMI | | | -0.00385 | | | | -0.0092, | | | | 0.00151 | | | |  |
| waist circumference | | | -0.000652 | | | | -0.00263, | | | | 0.00133 | | | |  |
| % body fat | | | 0.00251 | | | | -0.00193, | | | | 0.00695 | | | |  |
| Metabolic syndrome | | | -0.0248 | | | | -0.0549, | | | | 0.00529 | | | |  |
|  |  | | | |  | | | |  | | | |  |  |  |
| **Interactions with age 65 and above** | | | | |  | | | |  | | | |  |  |  |
|  | **Model 4 - demographic + health related behaviors + cell type adjusted** | | | | | | | | | | | |  |  |  |
|  | **coef** | | | | **95% CI** | | | | | | | |  |  |  |
| Lipoproteins | |  | | | |  | | | | |  | | | |  |
| HDL cholesterol | | 0.00107 | | | | -0.000875, | | | | | 0.00302 | | | |  |
| LDL cholesterol | | 0.00109 | | | | -0.00056, | | | | | 0.00274 | | | |  |
| Triglycerides | | -0.000103 | | | | -0.000697, | | | | | 0.00049 | | | |  |
| Blood sugar | |  | | | |  | | | | |  | | | |  |
| Glucose | | 0.000109 | | | | -0.00177, | | | | | 0.00198 | | | |  |
| Insulin resistance | | -0.00317 | | | | -0.0155, | | | | | 0.00913 | | | |  |
| HbA1c | | 0.00563 | | | | -0.0224, | | | | | 0.0336 | | | |  |
| Circulatory pressure | |  | | | |  | | | | |  | | | |  |
| Systolic blood pressure | | 0.000176 | | | | -0.00157, | | | | | 0.00192 | | | |  |
| Diastolic blood pressure | | 0.000248 | | | | -0.00196, | | | | | 0.00246 | | | |  |
| Pulse rate | | -0.00115 | | | | -0.00308, | | | | | 0.000782 | | | |  |
| Immune function | |  | | | |  | | | | |  | | | |  |
| C-reactive protein | | -0.00412 | | | | -0.0376, | | | | | 0.0294 | | | |  |
| Fibrinogin | | 0.0262 | | | | -0.0244, | | | | | 0.0768 | | | |  |
| Kidney function | |  | | | |  | | | | |  | | | |  |
| Cystatin C | | 0.0586 | | | | -0.0287, | | | | | 0.146 | | | |  |
| Glomelular filtration rate | | 0.00107 | | | | 0.0000132, | | | | | 0.00212 | | | |  |
| Albumin : Creatinine | | 0.0000619 | | | | -0.00000953, | | | | | 0.000133 | | | |  |
| Adiposity | |  | | | |  | | | | |  | | | |  |
| BMI | | 0.00161 | | | | -0.0048, | | | | | 0.00803 | | | |  |
| waist circumference | | -0.000469 | | | | -0.00279, | | | | | 0.00185 | | | |  |
| % body fat | | 0.00349 | | | | -0.00255, | | | | | 0.00952 | | | |  |
| Metabolic syndrome | | 0.000467 | | | | -0.0354, | | | | | 0.0364 | | | |  |

Model 4 adjusts for the following covariates: race/ethnicity (white, Mexican American, black and other), gender, foreign birthplace, education (less than high school, high school diploma, more than high school), class of work (White collar high, Blue collar high, White collar low, Blue collar low, no work), income, marital status (married or living with partner), age (as continuous), age-squared, white blood cells (SI), lymphocytes (%), monocytes (%), basophils (%), eosinophils (%), neutrophils (%), ever smoked, current smoker, moderate physical activity and vigorous physical activity. Coefficients are for the interaction between cardiovascular risk biomarker and the sociodemographic term indicated, where the indicated sociodemographic term takes on the value of 1 and all other levels of the variable are 0.
