# Supplementary material for: Leukocyte Telomere Length in Relation to 17 Biomarkers of Cardiovascular Disease Risk: A Cross-Sectional Study of US Adults
Source: PLoS Med. 2016 Nov 29;13(11):e1002188. doi: 10.1371/journal.pmed.1002188 (PMC5127504; doi:10.1371/journal.pmed.1002188)
Supplement: S5 Table — (DOCX) [file pmed.1002188.s006.docx]

|  | **Model 1 - unadjusted** | | | **Model 2 - demographic adjusted** | | |
| --- | --- | --- | --- | --- | --- | --- |
|  | **coef** | **95% CI** | | **coef** | **95% CI** | |
| Lipoproteins |  |  |  |  |  |  |
| HDL cholesterol | -0.0219 | -0.061, | 0.0171 | 0.0338 | -0.00795, | 0.0756 |
| LDL cholesterol | -0.0126 | -0.0378, | 0.0127 | 0.00491 | -0.0192, | 0.029 |
| Triglycerides | -0.0516 | -0.113, | 0.00977 | -0.0263 | -0.0825, | 0.03 |
| Blood sugar |  |  |  |  |  |  |
| Glucose | -0.0188 | -0.123, | 0.0858 | 0.052 | -0.0326, | 0.137 |
| Insulin resistance | 0.108 | 0.0118, | 0.205 | 0.0545 | -0.0403, | 0.149 |
| HbA1c | -0.0336 | -0.116, | 0.0489 | -0.0249 | -0.106, | 0.0559 |
| Circulatory pressure |  |  |  |  |  |  |
| Systolic blood pressure | -0.0815 | -0.135, | -0.0285 | -0.0132 | -0.0638, | 0.0375 |
| Diastolic blood pressure | -0.00663 | -0.0492, | 0.0359 | -0.035 | -0.0774, | 0.00749 |
| Pulse rate | 0.000131 | -0.0296, | 0.0299 | -0.0189 | -0.049, | 0.0112 |
| Immune function |  |  |  |  |  |  |
| C-reactive protein | 0.003 | -0.0638, | 0.0698 | -0.0207 | -0.0895, | 0.0481 |
| Fibrinogin | 0.00504 | -0.0576, | 0.0677 | 0.0283 | -0.0334, | 0.0901 |
| Kidney function |  |  |  |  |  |  |
| Cystatin C | -0.199 | -0.299, | -0.1 | -0.0585 | -0.153, | 0.036 |
| Glomelular filtration rate | 0.0487 | -0.00989, | 0.107 | -0.0301 | -0.0966, | 0.0363 |
| Albumin : Creatinine | 0.0194 | -0.022, | 0.0609 | -0.032 | -0.0898, | 0.0257 |
| Adiposity |  |  |  |  |  |  |
| BMI | 0.077 | -0.0000354, | 0.154 | -0.0302 | -0.109, | 0.0485 |
| waist circumference | -0.0918 | -0.167, | -0.017 | 0.013 | -0.0518, | 0.0778 |
| % body fat | -0.0392 | -0.0793, | 0.000949 | -0.0125 | -0.0557, | 0.0306 |

The effective sample size for each of the models was 3407 because of the use of fasting sample weights. Model 1 includes only the 17 biomarkers shown in the table. Model 2 additionally adjusts for the following covariates: race/ethnicity (white, Mexican American, black and other), gender, foreign birthplace, education (less than high school, high school diploma, more than high school), class of work (White collar high, Blue collar high, White collar low, Blue collar low, no work), income, marital status (married or living with partner), age (as continuous) and age-squared.
